# Supplementary material for: Motivation, inclusivity, and realism should drive data science education
Source: F1000Res. 2024 Apr 4;12:1240. Originally published 2023 Sep 28. [Version 2] doi: 10.12688/f1000research.134655.2 (PMC11101914; doi:10.12688/f1000research.134655.2)
Supplement: Peer review history

# Reviewer report: Reviewer response for version 2

**DOI:** 10.5256/f1000research.163287.r262947

**Published:** 2024-05-17

**Authors:** Johanna Hardin

**Affiliations:** 1 Pomona College, Claremont, California, USA

My thoughts on the paper are similar to those I had previously. My comments were not addressed or even engaged with. From my previous report: 

- It still seems odd to me that you leave out undergraduates as students who can be helped by the program. If you really want to leave them off, you might explain why you've made that deliberate decision "high school students, graduate students, postdocs, researchers, and university faculty".  (Especially as you note that GDSCN supports students from undergraduate institutions.)
- Reference 31 still seems like the wrong reference to me to support "just not good at it.

Minor:

 There are some places where a possible conversion has rendered words oddly, e.g., "re- searchers" "disser- vice"  (which I pointed out previously.)"

Is the topic of the opinion article discussed accurately in the context of the current literature?

Yes

Are arguments sufficiently supported by evidence from the published literature?

Yes

Are all factual statements correct and adequately supported by citations?

Yes

Are the conclusions drawn balanced and justified on the basis of the presented arguments?

Yes

Reviewer Expertise:

biostatistics, statistics and data science education

I confirm that I have read this submission and believe that I have an appropriate level of expertise to confirm that it is of an acceptable scientific standard.

# Reviewer report: Reviewer response for version 1

**DOI:** 10.5256/f1000research.147730.r214470

**Published:** 2023-11-27

**Authors:** Karen Word

**Affiliations:** 1 Software Carpentries, Oakland, USA

This is a well-informed article, presenting opinions that are grounded in attentive experience and consistent with evidence for best practices in training. It is helpfully organized and persuasive. I have no doubt that it will be a useful resource for anyone engaged in data science training. 

 I have several optional suggestions for improvement:

 1. The abstract cites 3 main ideals; the paper cites 4. I see no reason to omit iterative updating from the list in the abstract.

 2. The paper is nicely organized, with sections for each ideal, subheader topics relevant to each ideal, and "Practical ideas" for most of them. In some cases valuable examples are provided, illustrating how certain ideas have been applied in the authors' programs.  

- These examples are excellent and inspiring. Are there more of them? Consider featuring them more prominently e.g. in an "applied examples" section.
- "ideas" has an indefinite implication and isn't the word I would use. These seem to be solid, battle-tested recommendations. Maybe "Practical suggestions"?

3. The concept of growth mindset and the power of "yet" are both better attributed to Carol Dweck, though possibly in different sources. 

 4. Many items under inclusivity and realism explicitly relate to motivation. That's probably inevitable but it also stands out awkwardly - it might help to acknowledge that these ideals are often directly related.

 5. The "model making mistakes" bullet mentions "live coding" but I don't see a prior explanation of this term.

 6. In your ideas for being silly (wonderful!), you encourage using fun data examples. This is a good idea, but it is also a hazardous one, and I think that is worth a caveat that goes further than "as long as the data is appropriate." Good data sets have to meet needs of simplicity, complexity, availability, and ethics. Data sets that some people find "fun," in particular, may be upsetting for others (e.g. health or demographic data). Citing a resource on dataset selection may help here, or (better yet) you could expand on this issue separately under "inclusivity". (I'm sure there are other resources, but The Carpentries' lesson development training does have a section on this.)

 7. "Frequently take the temperature of the room" has a lot packed into it. Consider separating out one or two other bullets for emphasis on this vital topic? (And don't forget to account for color-blindness in post-it selection - green can be hard to find a good contrasting color for!)

 8. Under "Realism", the second paragraph mentions Bloom's taxonomy and "oversaturation." Neither of these is discussed or defined elsewhere. I'd suggest either fleshing this out or removing the jargon.

 9. Also under "Realism": could some of the introductory content more usefully be a "Practical ideas for making content relevant" set of bullets?

 10. Under "iteratively update" - this seems to overlook a major obstacle to succeeding at maintenance, despite good intentions: failing to plan for it. Specifically, to reserve time, assign responsibility, and structure procedures for this important and ongoing work. Maybe worth a mention?

 A few notes on resources (Full disclosure: I work for The Carpentries; sharing ours only in case they are useful):

 This paper cites an older version of The Carpentries Instructor Training Curriculum. That's fine, but if you'd like to point people at the most current version of this iteratively updated curriculum, the citation is attached (https://zenodo.org/records/5709383).

 Greg Wilson's book, Teaching Tech Together, is a separate but related resource that I would highly recommend exploring and citing.

 The "Curriculum Tools" page you attach cites an outdated version of The Carpentries lesson template. We believe the newer Workbench infrastructure & documentation offers a much more robust and accessible platform for developing curriculum on GitHub. 

 The Carpentries' Toolkit of IDEAs publication may also be useful in your list of IDARE resources (https://zenodo.org/records/7041935).

Is the topic of the opinion article discussed accurately in the context of the current literature?

Yes

Are arguments sufficiently supported by evidence from the published literature?

Partly

Are all factual statements correct and adequately supported by citations?

Yes

Are the conclusions drawn balanced and justified on the basis of the presented arguments?

Yes

Reviewer Expertise:

education, data science training, behavioral endocrinology, metabolic physiology

I confirm that I have read this submission and believe that I have an appropriate level of expertise to confirm that it is of an acceptable scientific standard.

## References

[1] : Mindset: The New Psychology of Success. *New York: Ballantine Books* .2008;

[2] : Teaching Tech Together.2019; 10.1201/9780429330704 10.1201/9780429330704

[3] : sandpaper: Create and Curate Carpentries Lessons. *Carpentries* .2023; Reference source

# Reviewer report: Reviewer response for version 1

**DOI:** 10.5256/f1000research.147730.r214465

**Published:** 2023-11-27

**Authors:** Johanna Hardin

**Affiliations:** 1 Pomona College, Claremont, California, USA

The article provides direction for data science educators to teach more effectively. In particular, the authors speak to creating space so that all learners feel welcomed in and compelled to participate in the larger data science community.  The authors suggest that the way to engage learners who have traditionally been kept out of data science is through motivation, inclusivity, and realism. While their work builds on scholarship that has already given very similar perspectives, the innovation is in the presentation of the direct suggestions, particularly the practical tips given within each section which are excellent.

 The paper is well-presented and generally easy to engage with. However, I have a few suggestions that might make reading the paper slightly easier.

 1. I'm slightly confused about the background programs of the authors. What is the goal of communicating the projects?  It seems like DataTrail is the main one referenced throughout the paper. Additionally, the authors say things like "For more about good documentation you can see our course.49" with no suggestion to the reader that you are referring to ITN. Further clarification on the links between the program and the ideas would be welcome.

 2. I might suggest adding "Create a reproducible example" (using reprex?) as another practical idea for imparting good communication skills.

 3. You say "Our lab is involved in a number of data science education efforts, including training high school students, graduate students, postdocs" ... is it really true that none of you have ever worked with undergraduates?

 4. You say "Learning data science is hard. This means learners tend to believe that they are “just not good at it" and that this is a fixed intrinsic quality they possess. These beliefs can affect not only a student’s confidence but perhaps worse, a teacher’s behavior.31"  But reference 31 is about gender stereotypes and whether students persist in STEM. I'm not sure it is about "just not good at it" or a teacher's behavior. I didn't check all of the references.

 5.  There are a number of phrases with awkward wording:

- Our program goes beyond programming (pg 4)
- There are over 10 case studies that are currently focused on utilizing methods in R, statistics, data science, and public health to evaluate timely and relevant public health questions. (pg 4)
- involves a lot of questions and failure and that is good (pg6)
- We do learners a disser- vice (pg 9)
- The example code that you use to teach should self-explanatory. (pg 12)
- We would also like to acknowledge the students we have taught, who continue to inspire us to be better educators and provided valuable and helpful feedback. (pg 14)

6. Of particular note is the 100+ times that the phrases this/these are used. I might suggest that the authors read through the manuscript and see if the language can be tightened up by removing their use. For example, "formal Master’s programs and certificates in data science indicate the great demand for these materials...While data science instructors can access these excellent and inspiring training materials."  What materials?

 7. The summary page of practical tips (very end, pg 14) says that the link will take the reader to a PDF, but it goes to a markdown file on GitHub.

 8. Is it important to call out computational biology in your first sentence? Why?

Is the topic of the opinion article discussed accurately in the context of the current literature?

Yes

Are arguments sufficiently supported by evidence from the published literature?

Yes

Are all factual statements correct and adequately supported by citations?

Yes

Are the conclusions drawn balanced and justified on the basis of the presented arguments?

Yes

Reviewer Expertise:

biostatistics, statistics and data science education

I confirm that I have read this submission and believe that I have an appropriate level of expertise to confirm that it is of an acceptable scientific standard, however I have significant reservations, as outlined above.
